# Sanitary Legislation and Administration
*Sanitary Legislation and Administration. An Address. By Henry Wyldbore Rumsey. London: Churchill. 1858.


**Published:** 1858-04

**Authors:** 


### THE EPITOME OF SANITARY LITERATURE.

### SANITARY LEGISLATION AND ADMINISTRATION.*

In this address, Mr. Rumsey reproduces certain of his particular views regarding sanitary legislation. Our readers are already informed as to the manner by which Mr. Rumsey proposes to make this country the paradise of health. They will share with us in our admiration of the enthusiasm and earnestness of the projector, while they look doubtfully on the lines which mark the projection. We presume, however, from the present work, that Mr. Rumsey himself is modifying in his own mind the comprehensive plan for State interference, which he propounded in his essays on State Medicine. On the present occasion he tells us what education, *minus* law, may perform. While talking of comprehensive legislation, he urges that although it would not be desirable to legislate upon all sanitary matters in one session or two, yet "every instalment" of sanitary reform should be part of *one* large and statesman-like project, "so that as the several measures are produced, one by one in judicious succession, when the public mind is prepared for them, they may fit harmoniously together, like the architectural details of some vast pile, planned at one time though reared at intervals, and when completed exhibiting unity of design, and standing in simple grandeur an object for the admiration of mankind."

Mr. Rumsey still opposes (and in this we are not at variance with him) local sanitary legislation. What, he asks, would have been the result of limiting the Poor Law Amendment Act to parishes petitioning for its adoption? He quotes Chalmers, to the effect, that opposition to measures of improvement would in all probability proceed from the very places which most needed those measures. The demand for exemption would be the proof of their necessity. Surely then, thus runs the argument, the application of general hygienic laws ought to be the very last duty of local administration.

But while inclined to modify some of his more extreme views, as to the wisdom, nearly divine, of State intellect, and as to the force, more than mortal, which such surpassing intelligence should put forth for sanitary jurisdiction, the old spirit which entered so largely into the State Medicine essays is still traceable. *Vide*

"It would be waste of words to argue with those who, disgusted with our puny and abortive attempts at sanitary legislation, hastily

---

* Sanitary Legislation and Administration. An Address. By Henry Wyldbore Rumsey. London: Churchill. 1858.

exclaim against any state intervention, any comprehensive code of public health,—who imagine that all may be left to the wider diffusion of knowledge, and to the unguided exercise of the individual will; but who thus shut their eyes to the history of man, who forget the natural tendency of our race to social degradation—to mental and bodily deterioration, when not upheld by the divine principle of order, and who ignore the proved fact that law and government are indispensable to civilisation. My business is not with such speculators. They must, if consistent, call for the abrogation of every sanitary law in the statute book."

There is nothing more remarkable than the observation how far extreme enthusiasm in one direction will lead the earnest writer. Think, now, that any one should rise and proclaim that the first of the life beings whom the Supreme has led to this earth, has but one natural tendency—*to degrade*, to descend toward the lower tribe of animal. In the name of our common humanity and the grand purposes for which we are each and all formed, we meet this speculative statement with flat opposition, as alike contrary to the natural and divine will and to the facts of history. We affirm, that in the light and radiance of knowledge man, individually and collectively, aspires to the highest position in civilisation; and we repeat what has been said by the personation of the first intellect that has yet visited this planet,—

"Ignorance is the curse of God;
Knowledge the wing on which we fly to heaven."

Again, the idea of trusting to the wider diffusion of knowledge and to the unguided exercise of the individual will, includes, as Mr. Rumsey puts it, a contradiction. If knowledge is widely diffused, the individual will is not unguided, but guided by the knowledge, the strongest of all guides; while the assertion of "the proved fact, that law and government are indispensable to civilisation," embraces only an assumption. That law and government have been coincident with progress is a necessary historical fact. It is conceded; but in the aggregate of history no more is told. Some laws have, truly, by their wisdom helped progress and civilisation; other laws and governments have held back progress for ages, damned knowledge, and blackened humanity. Were the laws against witches indispensable to civilisation? Have the laws which gave origin to the public executioner been indispensable to civilisation? It is a delicate question forsooth how far law, in the main, has influenced mankind for good or for evil. But this is clear, that as laws are out of men and through men, every law can only denote the degree of advancement to which men had attained at the time when such law was made. *Ergo*, the laws are a sequence. If civilisation is advanced, the laws are indices of civilisation. If the age is gross and barbarous, its laws are like it.

We have so often pointed out what are the legitimate functions of government in reference to sanitary legislation, that we do not dwell on this topic. We believe, with Mr. Rumsey, that a sanitary council of State is advisable. We would give to such council the highest honours. We would give to it certain limited powers for great emergencies. We would relieve it of the ignorant interference of local health legislating amateurs ; but we would confine its duties mainly to investigation, recommendation and instruction, on the broad principle that to know the worst is to secure the best. As to the taunt which Mr. Rumsey throws out, that men who hold the opinions we now respectfully offer "must, if consistent, call for the abrogation of every sanitary law on the statute book," we, for our parts, prepared to be consistent, bear it lightly. For as there is not a pure sanitary law in the statute book which in any way comprehensively touches for good any sanitary point, we think, that the sooner the State porridge is strained from its chips, the better for the State.

We cannot, in conclusion, but regret that so able and eminent a sanitarian as Mr. Rumsey should employ his time and thoughts in the wildest speculative schemes of impossible legislation, when he might, by simple teaching and simple scientific investigation, perform in deed that which he now comprises in mere utterances. In the present position of the political forces of England there is, we boldly affirm, an entire want of every element for the construction of a philosophical scheme of sanitary legislation, granting even such scheme desirable. We assert further, that by the time the political element for such philosophical legislation is found, it will not be wanted ; since a people, who are so enlightened as to bring together the philosophical law-makers, will have learnt the philosophy for themselves, and taught it to their children, without the aid of either statute-book or statute.

